# An update on non-invasive urine diagnostics for human-infecting parasitic helminths: what more could be done and how?

**DOI:** 10.1017/S0031182019001732

**Published:** 2020-07

**Authors:** John Archer, James E. LaCourse, Bonnie L. Webster, J. Russell. Stothard

**Affiliations:** 1 Wolfson Wellcome Biomedical Laboratories, Department of Zoology, Natural History Museum, Cromwell Road, London SW7 5BD, UK; 2 Department of Tropical Disease Biology, Liverpool School of Tropical Medicine, Pembroke Place, Liverpool L3 5QA, UK.

**Keywords:** Helminth diagnosis, helminthiases, non-invasive urine sampling, urine diagnostics

## Abstract

Reliable diagnosis of human helminth infection(s) is essential for ongoing disease surveillance and disease elimination. Current WHO-recommended diagnostic assays are unreliable in low-endemic near-elimination settings and typically involve the invasive, onerous and potentially hazardous sampling of bodily fluids such as stool and blood, as well as tissue *via* biopsy. In contrast, diagnosis by use of non-invasive urine sampling is generally painless, more convenient and low risk. It negates the need for specialist staff, can usually be obtained immediately upon request and is better accepted by patients. In some instances, urine-based diagnostic assays have also been shown to provide a more reliable diagnosis of infection when compared to traditional methods that require alternative and more invasive bodily samples, particularly in low-endemicity settings. Given these relative benefits, we identify and review current research literature to evaluate whether non-invasive urine sampling is currently exploited to its full potential in the development of diagnostic tools for human helminthiases. Though further development, assessment and validation are needed before their routine use in control programmes, low-cost, rapid and reliable assays capable of detecting transrenal helminth-derived antigens and cell-free DNA show excellent promise for future use at the point-of-care in high-, medium- and even low-endemicity elimination settings.

## Introduction

**Fig. 1.**
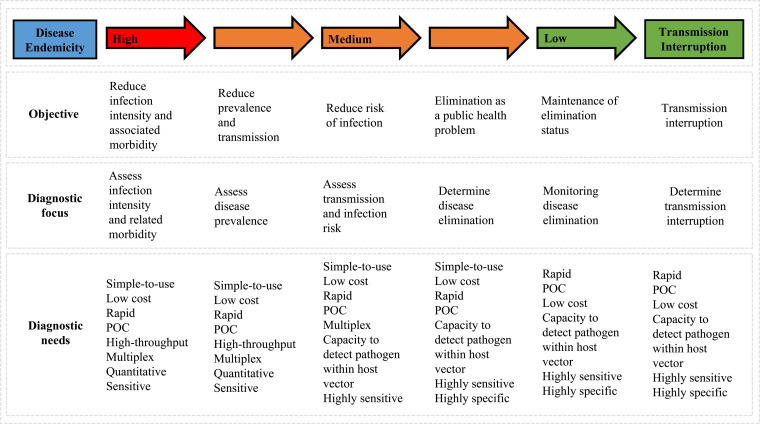
Schematic outlining changes in diagnostic priorities as control programmes progress (adapted from Bergquist *et al*., 2009).

Parasitic worms, often referred to as helminths, form the most common human infectious parasites in low- and middle-income countries (LMICs), causing a global burden of disease exceeding that of both malaria and tuberculosis (Hotez *et al*., 2008; Lustigman *et al*., 2012). The rapid, straightforward and reliable diagnosis of helminthiases is essential for ongoing disease surveillance and successful disease control, particularly as control programmes advance towards disease elimination within endemic areas (Fig. 1), (Bergquist *et al*., 2009; Gordon *et al*., 2011; McCarthy *et al*., 2012; Rollinson *et al*., 2013; Werkman *et al*., 2018).

**Fig. A1.**
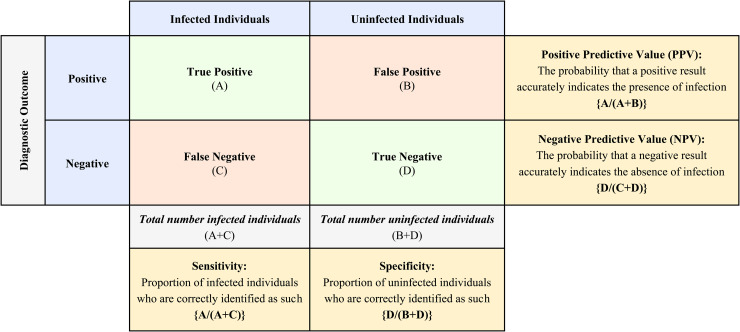
Defining sensitivity, specificity and predictive values (Akobeng, 2007; Bergquist *et al*., 2009).

Current ‘gold standard’ diagnostic assays for the majority of these diseases typically involve the invasive and cumbersome sampling of bodily fluids such as stool and blood, as well as tissue *via* biopsy (Table 1), (WHO, 2012). Not only are these procedures often painful, onerous and carry a risk of infection (with, for example, HIV), but they also require specific equipment and specialist health workers seldom available in endemic areas. A reliable assessment of disease prevalence within a given community can therefore often prove challenging as a result of patient aversions to being assessed, as well as through a lack of resources (Itoh *et al*., 2011). Although widely considered low-cost, when taking into consideration the cumulative costs of equipment, number of personnel needed and remuneration of specialist staff, the true costs of gold standard assays are also being realized now and may likely be far more expensive than previously assumed (Turner *et al*., 2017). In addition, whilst these techniques may be sufficiently sensitive to confirm or refute individual infection status in areas of high disease endemicity or when assessing patients burdened with a high degree of infection, in areas of low-endemicity, for example during control programme near-elimination settings, sensitivity of these techniques can seriously wane (Appendix Fig. A1), (Bergquist *et al*., 2009; Klepac *et al*., 2013; Hawkins *et al*., 2016).

**Table 1.** WHO-recommended diagnostic techniques for major human helminth infections and how technique invasiveness compares to that of urine sampling.

Disease (also known as)	Infectious agent (helminth species)	WHO-recommended diagnostic technique (WHO, 2012)	Degree of sample invasiveness relative to urine sampling*
Urogenital Schistosomiasis (Bilharzia/Snail Fever)	*Schistosoma haematobium*	Identification of ova in concentrated urine sample *via* microscopy	±
Ascariasis (Roundworm)	*Ascaris lumbricoides*	Identification of ova in concentrated faecal smear *via* microscopy	+
Trichuriasis (Whipworm)	*Trichuris trichiura*		+
Hookworm Infection	*Ancylostoma duodenale*		+
	*Necator americanus*		+
Gastrointestinal Schistosomiasis (Bilharzia/Snail Fever)	*Schistosoma mansoni*		+
	*Schistosoma japonicum*		+
	*Schistosoma mekongi*		+
	*Schistosoma guineensis*		+
	*Schistosoma intercalatum*		+
Liver Fluke Infection	*Fasciola hepatica*		+
	*Fasciola gigantica*		+
	*Opisthorchis viverrini*		+
Taeniasis (Tapeworm infection)	*Taenia solium* (adult stage)		+
	*Taenia saginata* (adult stage)		+
Strongyloidiasis (Threadworm infection)	*Strongyloides stercoralis*	Identification of larvae in concentrated faecal smear *via* microscopy	+
Lymphatic Filariasis (Elephantiasis)	*Wuchereria bancrofti*	Identification of microfilariae in blood smear (taken to coincide with blood-circulating periodicity behaviour) *via* microscopy	++
	*Brugia malayi*		++
	*Brugia timori*		++
Loiasis (Loa)	*Loa loa*	Identification of microfilariae in blood smear *via* microscopy	++
Cysticercosis/neurocysticercosis	*Taenia solium* (larval cysts)	MRI or CT brain scan	++
Onchocerciasis (River Blindness)	*Onchocerca volvulus*	Identification of microfilariae in multiple skin snips *via* microscopy	+++

*Positive/negative symbols denote degree of increase in sample invasiveness when compared to urine sampling where: ‘±’ indicates relative comparable invasiveness; ‘+’ indicates a moderate increase in sample invasiveness; ‘++’ indicates a considerable increase in sample invasiveness and; ‘+++’ indicates a major increase in sample invasiveness.

In contrast, diagnosis by use of non-invasive urine sampling is generally painless, more convenient, less expensive and low risk. It negates the need for specialist staff, can usually be obtained immediately upon request and is better accepted by patients (Castillo *et al*., 2009). Further to these clear practical advantages, some urine-based diagnostic assays have also been shown to provide a more sensitive diagnosis of infection when compared to traditional methods that require alternative and more invasive bodily samples, particularly in low-endemicity settings (Sousa-Figueiredo *et al*., 2013; Adriko *et al*., 2014). Given these relative benefits in ease of collection, greater patient acceptability and possible improved diagnostic performance, the following review aims to evaluate whether urine is currently being exploited to its full potential with regards to the diagnosis of the major human helminth infections and highlight future research needed to further advance helminth urine-diagnostics.

## Literature search strategy

A systematic online literature search was conducted, beginning in October of 2018 and ending in October of 2019. The PubMed, Cochrane Library, Google Scholar and Web of Science databases were used, following stipulated database guidelines, to search for any literature published between 1919 and 2019 within peer-reviewed journals relevant to inputted search terms (National Center for Biotechnology Information., 2019; Cochrane Library., 2019; Google Scholar., 2019; Web of Science., 2019).

Three focal search terms, ‘diagnosis’, ‘diagnostic’ and ‘detection’, were used in conjunction with either disease name(s) (e.g. ‘schistosomiasis’, ‘Bilharzia’ and ‘snail fever’ or ‘lymphatic filariasis’ and ‘elephantiasis’), or pathogen species (e.g. ‘*Schistosoma haematobium*’ or ‘*Wuchereria bancrofti*’) and ‘urine’ or ‘transrenal’. Following this initial search, additional terms were included, such as diagnostic marker (e.g. ‘antigen’) and/or assay method (e.g. ‘enzyme-linked immunosorbent assay’), to potentially uncover additional literature. The abstracts of all publication hits were read and assessed for their relevance to review. Irrelevant articles were not included in the review, whereas all relevant articles were read in full. Publications deemed relevant were those that highlighted any primary research concerning the detection of any human-infecting parasitic helminth outlined in Table 1, or closely related non-human animal-infecting species, within urine samples taken from humans or non-human animals. Any secondary research, for example, systematic reviews or meta-analyses that met these criteria were also included. All literature cited within relevant articles was also screened, again to potentially uncover additional literature not provided by initial database searches.

## Macroscopic changes to urine as a means of diagnosing urogenital schistosomiasis

Visible haematuria is often indicative of active urogenital schistosomiasis, caused by infection with *Schistosoma haematobium* (Colley *et al*., 2014). As such, cost-effective questionnaires involving either the self-reporting of blood in the urine by patients or the observation of blood in the urine by health workers have been used in an attempt to rapidly identify infected individuals and disease prevalence within endemic areas (Lengeler *et al*., 2002*a*,*b*; Okeke and Ubachukwu, 2014; Atalabi *et al*., 2017).

The sensitivity of self-reporting the presence of blood in the urine for diagnosis of *S. haematobium* infection has been extensively assessed (Bogoch *et al*., 2012; Bassiouny *et al*., 2014). Comparing patient questionnaire responses to the diagnostic gold standard (identification of ova in concentrated urine samples *via* microscopy), it has been concluded that despite the method's practical advantages and relatively low cost, self-reported macrohaematuria alone is unreliable at the individual level primarily because visible haematuria typically only presents in individuals burdened with particularly heavy infections (Bogoch *et al*., 2012). In addition, macrohaematuria is also often a symptom of common urinary tract infections and bladder stones (Appendix Fig. A1), (Le and Hsieh, 2017). It has also been highlighted that the self-reporting of blood in the urine by school-aged children, the demographic customarily selected for helminth surveillance within a given community, can be unreliable due to either a young girl's reluctance to admit the onset of menses, or a young boy's eagerness to proclaim his ‘coming of age’ as a result of gross haematuria often being considered a natural sign of the onset of puberty (Montresor *et al*., 2002; Colley *et al*., 2014).

For these reasons, the diagnostic reliability of having trained and experienced personnel to identify the presence of macroscopic blood in the urine has also been assessed, again, comparing the method to urine-egg detection by microscopy (Okeke and Ubachukwu, 2014). Once more it was concluded that, unless used in conjunction with more taxing and costly methods, macrohematuria does not provide adequate sensitivity when compared to egg microscopy, and in using only this method low-, or even moderate-intensity, infections would likely be missed.

It is generally accepted that although a useful and easily implemented tool in initial baseline observations to confirm *S. haematobium* presence in highly-endemic populations, in areas of low-endemicity, or when evaluating programmatic intervention success in reducing disease prevalence and transmission, alternative and more accurate diagnostic approaches should be used (Utzinger *et al*., 2015; Mutapi *et al*., 2017).

## Microscopic changes to urine as a means of diagnosing urogenital schistosomiasis

The current gold standard of *S. haematobium* diagnosis involves the filtering, staining and observation of morphologically distinct eggs excreted in urine (Le and Hsieh, 2017). Using a syringe and polycarbonate filters with a pore size of 8–30 *μ*m, eggs from 10 mL of a well-shaken urine sample can be isolated, stained and examined under a microscope (Peters *et al*., 1976; Colley *et al*., 2014; Utzinger *et al*., 2015). This has long been the preferred method of *S. haematobium* diagnosis as it allows for a straightforward and reasonably inexpensive means of confirming infection within an individual or presence within a community (through sample pooling), using relatively unsophisticated and somewhat field-appropriate equipment. Additionally, and importantly, eggs can be quantified; providing a moderately accurate assessment of infection intensity within an individual that can then be used to estimate the degree of clinical morbidity (Colley *et al*., 2014; Utzinger *et al*., 2015; Corstjens *et al*., 2017).

The many shortcomings of urine-egg microscopy, however, are well understood (Braun-Munzinger and Southgate, 1992; Le and Hsieh, 2017; Ajibola *et al*., 2018). Owing to heterogeneities in egg output occurring between different periods of the same day, between different days and even between different seasons, accurate diagnosis and morbidity assessment of any given individual using just one urine sample is unlikely (Braun-Munzinger and Southgate, 1992; Le and Hsieh, 2017; Christensen *et al*., 2018). To mitigate this, multiple urine samples from the same individual can be taken over consecutive days, ideally between the hours of 10:00am and 2:00pm to coincide with optimum egg passage (Le and Hsieh, 2017). Repeated bouts of urine filtration and microscopy is, however, taxing work; a reasonable balance between diagnostic accuracy, time spent and financial cost must be met and even then, overt improvements in diagnostic sensitivity are rarely seen (Stothard *et al*., 2014). Differences in diagnostic sensitivity between more and lesser-experienced technicians are also often found, further complicating matters when large quantities of urine samples require assessment (Knopp *et al*., 2015).

Of more urgent concern is urine-egg microscopy's poor sensitivity when used in areas of low- or even moderate-prevalence settings (WHO, 2013; Le and Hsieh, 2017). As egg output declines, the sensitivity of urine-egg microscopy is significantly reduced resulting in a variety of challenges beyond just reliably identifying individuals burdened with low-intensity infections. Some of these challenges include accurately estimating clinical morbidity, evaluating the impact of programmatic interventions, diagnosing pre-school aged children and assessing new diagnostic tools (Stete *et al*., 2012; Knopp *et al*., 2013, 2018; Le and Hsieh, 2017). Recent concern has also been raised about urine-egg microscopy's poor sensitivity when attempting to detect ‘ultra-light’ infections, regarded as those that result in the expulsion of between only one and five eggs per 10 mL of urine (Knopp *et al*., 2018). Given the reproductive biology of schistosomes, just one infected individual excreting such minute numbers of eggs that may go on to infect and asexually reproduce within the appropriate intermediate freshwater snail host, potentially producing hundreds of cercariae per day, can cause the re-infection of an entire community (Colley *et al*., 2014). As such, in elimination settings or where treatment is targeted only to infected individuals that may be tracked, reassessed and retreated, any infected individuals must be quickly identified to ensure prompt treatment and total interruption of transmission; highlighting the urgent need for rapid, simple-to-use diagnostic tools deployable at the point-of-care (POC) and able to detect ultra-light infections (Hawkins *et al*., 2016; Knopp *et al*., 2018).

Although macrohematuria is typically present only in those harbouring heavy *S. haematobium* infections, microhaematuria, i.e. trace amounts of blood in the urine not visible to the naked-eye, can occur even in moderate- and low-intensity infections and can be detected using rapid, simple-to-use and relatively inexpensive reagent-strips that can be used at the point-of-care (Ochodo *et al*., 2015; Le and Hsieh, 2017; Knopp *et al*., 2018).

The accuracy of urine-heme reagent-strips, or ‘dipsticks’ for the indirect diagnosis of urogenital schistosomiasis have also been extensively assessed (Robinson *et al*., 2009; Krauth *et al*., 2015; Hassan *et al*., 2018; Musa and Dadah, 2018; Knopp *et al*., 2018). Recent reviews and meta-analyses have been undertaken to evaluate their diagnostic accuracy with a specific focus on high-, medium- and low-prevalence settings and in populations that have previously undergone repeated mass drug administration (MDA) treatment with praziquantel (King and Bertsch, 2013; Ochodo *et al*., 2015). In most cases, it has been concluded that although the diagnostic performance of urine-heme dipsticks is reduced in low-transmission areas and despite a range of possible confounding reasons for the presence of blood in the urine (such as urinary tract infections, bladder stones and menstrual blood), at the population level, urine-heme dipsticks should be considered more accurate than urine-egg microscopy (Ochodo *et al*., 2015). In addition, urine-heme dipsticks do not require specially trained microscopists, are less influenced by daily fluctuations in egg passage and take far less time to carry out (Krauth *et al*., 2015). It has also been concluded, however, that whilst urine-heme dipsticks should continue to be used to monitor the early-stage population-level impact of schistosomiasis control programmes (i.e. when assessing the initial baseline prevalence or when evaluating changes in overall prevalence after early intervention implementation), in elimination settings, or again when treatment is targeted only to infected individuals, neither the urine-heme dipstick or urine-egg microscopy can reliably identify individuals burdened with low- or ultra-light-intensity infections still capable of maintaining disease transmission (King and Bertsch, 2013; Ochodo *et al*., 2015; Knopp *et al*., 2018). Further assessment of urine-heme dipstick diagnostic performance using more sophisticated and sensitive methods such as *Schistosoma* antigen or DNA detection, rather than egg microscopy, has been encouraged (King and Bertsch, 2013).

As well as microhaematuria, leukocyturia (the abnormal presence of white blood cells in the urine) and proteinuria (the abnormal presence of proteins in the urine) may also be used as proxy to diagnose urogenital schistosomiasis, though both methods been found to be significantly less sensitive and specific than urine-heme dipsticks (Ochodo *et al*., 2015). It has been suggested, however, that the use of urine-heme dipsticks in conjunction with low-cost and field-deployable assays capable of detecting albuminuria (urine-albumin concentrations of >40 mg L^−1^), may provide a reliable diagnosis of infection in high-endemicity settings whilst also allowing assessment of kidney and urinary tract morbidity associated with chronic disease (Rollinson *et al*., 2005; Sousa-Figueiredo *et al*., 2009).

Like macroscopic changes, microscopic changes to urine are also considered now insufficiently sensitive to detect *S. haematobium* infection in low-prevalence settings or within individuals harbouring low-level infections. In addition, these changes only occur as a result of infection with *S. haematobium*. In endemic areas, co-infection with multiple helminth species is commonplace, highlighting the need for multiplex assays capable of reliably detecting multiple helminth species using just one bodily sample.

## Detection of anti-helminth urine-antibodies

Immunodiagnostic assays for the detection of blood-circulating anti-helminth antibodies have been used to diagnose infection with many human-infecting helminthiases (Rebollo and Bockarie, 2014; Kemal *et al*., 2015; Vlaminck *et al*., 2015; Akue *et al*., 2018). Of all antibody-targeting immunological assays, the most frequently employed is some form of the enzyme-linked immunosorbent assay (ELISA), the diagnostic functionality of which relies on the highly specific antigen-antibody binding that occurs during the body's immune response to invading foreign pathogens (Lazcka *et al*., 2007).

Due to ease of sample procurement relative to blood sampling, the diagnostic potential of targeting anti-helminth antibodies expelled within the urine using immunodiagnostic assays has also been assessed; targeting and successfully detecting urine-based antibodies formulated against a range of helminth species (Table 2). Of those studies comparing the diagnostic accuracy between targeting urine- and serum-based antibodies, all reported good association in diagnostic performance whilst no additional effort in urine-sample preparation was required, presenting a compelling argument for moving beyond invasive blood-based diagnostics (Elhag *et al*., 2011; Nagaoka *et al*., 2013; Eamudomkarn *et al*., 2018).

**Table 2.** Anti-helminth antibodies detected within urine and immunodiagnostic assay used.

Bodily habitat	Species (life stage)	Antibody detected	Assay used	References
Circulatory system	*S. mansoni* (adult stage)	IgG against soluble worm antigen (SWA)	ELISA	(Elhag *et al*., 2011)
	*S. haematobium* (adult stage)	IgG against soluble worm antigen (SWA)	ELISA	(Elhag *et al*., 2011)
	*S. haematobium* (ova)	IgG against *S. haematobium* soluble egg antigen (SEA)	RDT-sh	(Sheele *et al*., 2013)
	*S. japonicum* (ova)	IgG against *S. japonicum* soluble egg antigen (SEA)	ELISA	(Itoh *et al*., 2003*a*)
Lymphatic system (adult stage/L3 larval stage) and/or circulatory system (microfilariae larval stage)	*W. bancrofti* (life- stage not specified)	Urinary IgG4 against *Brugia pahangi* crude soluble antigen	Modified ELISA: high-density latex bead assay	(Nagaoka *et al*., 2013)
			ELISA	(Itoh *et al*., 2003*b*; Weerasooriya *et al*., 2008)
		Filaria-specific IgG4	ELISA	(Rattanaxay *et al*., 2001; Itoh *et al*., 2007; Samad *et al*., 2013)
Gastrointestinal tract (adult stage) and/or circulatory system (larval stage)	*S. stercoralis* (life- stage not specified)	IgG against *S. ratti* crude soluble antigen	ELISA	(Eamudomkarn *et al*., 2018)
Liver	*O. viverrini* (adult stage)	IgG, IgA, IgG4 against *O. viverrini* crude somatic antigen	ELISA	(Sawangsoda *et al*., 2012)
		IgG, IgG4 against *O. viverrini* crude somatic antigen	ELISA	(Tesana *et al*., 2007)

Although highly specific even in low-endemicity settings, antibody detection using the ELISA requires sophisticated equipment, specially trained health workers and expensive reagents that require cold chain typically unavailable to those in disease-endemic regions, particularly when hoping to obtain a quantitative diagnosis that indicates degree of infection within an individual (Bergquist *et al*., 2009; Tchuem Tchuenté, 2011). As such, regardless of the bodily sample taken, these requirements make it difficult to envisage the future scale-up and field-deployment of the ELISA at the point-of-care, where the simple-to-use, rapid and sensitive diagnosis is needed. It is for this reason that much attention has been given to the development of simple-to-use point-of-care rapid diagnostic test (POC-RDT) devices capable of rapidly detecting blood-circulating anti-helminth antibodies (Weil *et al*., 2000; Coulibaly *et al*., 2013; Steel *et al*., 2013). Further development of these for use with urine samples, however, is lacking. Two novel transrenal antibody-detecting RDTs that have been developed and assessed involve the use of antigen-coated coloured latex beads for the detection of filaria-specific IgG4 (Nagaoka *et al*., 2013), and the filtering of urine to isolate human IgG bound to *S. haemotobium* ova, both requiring significantly less equipment, reagents and technical expertise than conventional immunodiagnostic assays (Sheele *et al*., 2013). Although promising, further evaluation for reliability, field-applicability, upscale and deployment is needed.

Another principal concern when targeting antibodies to determine infection status is the inability to distinguish between active and past infections owing to high antibody titres remaining within the body long after treatment success and infection clearance (Rollinson *et al*., 2013; Utzinger *et al*., 2015). This becomes particularly problematic when attempting to evaluate the impact of programmatic control strategies in areas that have undergone control intervention. As an example, individuals within areas having undergone mass administration with albendazole for the treatment of ascariasis may have indeed cleared any infection, however, any diagnostic assay targeting anti-*Ascaris* antibodies used to assess these individuals may remain positive (Jourdan *et al*., 2018). In areas where disease elimination is sought, it has been suggested that antibody-targeting assays may be appropriate for use with young children who have not yet received treatment as a means of assessing whether the transmission is still taking place (Jourdan *et al*., 2018; Takagi *et al*., 2019). In doing so, seroconversion rate, typically somewhere between at least 4 and 8 weeks after initial exposure, must be taken into consideration (van Grootveld *et al*., 2018; Vlaminck *et al*., 2019).

Persistent post-infection circulating antibodies also cause difficulty when attempting to evaluate the true accuracy of antibody-targeting diagnostic assays; typically performed *via* comparison to gold standard assays that may themselves have poor-sensitivity. In doing this, antibody assays will consistently appear highly-sensitive with likely concurrent low positive predictive values (PPV), (Appendix Figure A1), whereas individuals testing negative by gold standard methods but positive by antibody-detecting methods may plausibly be harbouring active but low-level infections, or may indeed be currently uninfected after having cleared a previous infection (Doenhoff *et al*., 2004).

Cross-reactivity of antibodies between different helminth genera is also an issue (Genta, 1988; Lammie *et al*., 2004; Weerakoon *et al*., 2015; Lamberton and Jourdan, 2015; Garcia *et al*., 2018; Song *et al*., 2018). In some cases, genera-, or even species-specific identification of infecting helminths is essential for safe treatment strategies, for example when providing ivermectin to treat onchocerciasis in loiasis-endemic areas (Gardon *et al*., 1997), or for diagnosis of species-specific pathologies such as female and male genital schistosomiasis (Itoh *et al*., 2011; Vlaminck *et al*., 2016; Kayuni *et al*., 2019; Kukula *et al*., 2019). In circumstances such as these, diagnostic assays with a higher degree of specificity than that of antibody-targeting assays are needed.

Because of the technical, financial and logistical challenges presented by anti-helminth antibody detection and when considering the very limited resources available for the development and validation of novel diagnostic assays, perhaps focus is best placed elsewhere, on more user-friendly, cost-effective and reliable methods.

## Detection of helminth-derived urine-antigens

Targeting urine-antigens has multiple advantages over targeting transrenal antibodies; detection of antigens indicates active infection; diagnostic assays that target antigens can, therefore, be used to evaluate disease intervention strategies such as MDA and vector control; invading parasites may be detected soon after infection and antigen levels generally correlate well with parasite load (Corstjens *et al*., 2014; Worasith *et al*., 2015; Ochodo *et al*., 2015; Kamel *et al*., 2019; Sousa *et al*., 2019). As with antibody detection, good association between urine- and serum-based antigen detection has been found in high-, medium- and low-endemicity settings, further strengthening the argument for moving towards non-invasive urine sampling (van Dam *et al*., 2004; Kamel *et al*., 2019; Sousa *et al*., 2019).

Again, most immunodiagnostic assays used to detect helminth-derived urine-antigens, such as conventional ELISAs, are currently unsuited for point-of-care use (Table 3). At present, efforts to develop simple-to-use POC-RDTs for the detection of helminth urine-antigens have focused primarily on test devices capable of diagnosing urogenital and intestinal schistosomiasis, though a point-of-care lateral-flow dipstick to detect *O. volvulus-*derived urine-antigens has also been developed (Ayong *et al*., 2005).

**Table 3.** Helminth-derived antigens detected within the urine and immunodiagnostic assay used.

Bodily habitat	Species (life stage)	Antigen detected	Assay used	References
Circulatory system	*S. mansoni* (adult stage)	Circulating cathodic antigen (CCA)	ELISA	(Ochodo *et al*., 2015; Peralta and Cavalcanti, 2018)
			CCA lateral-flow strip POC-RDT	(Stothard *et al*., 2006; Ochodo *et al*., 2015; Peralta and Cavalcanti, 2018)
		Circulating anodic antigen (CAA)	ELISA	(Peralta and Cavalcanti, 2018)
			Up converted phosphor-lateral flow CAA assay	(Sousa *et al*., 2019)
		Circulating *Schistosoma mansoni* antigen (CSA)	Lateral-flow immunochromatographic test strip/ELISA	(Kamel *et al*., 2019)
	*S. haematobium* (adult stage)	Circulating cathodic antigen (CCA)	ELISA	(Stothard *et al*., 2006; Ochodo *et al*., 2015; Peralta and Cavalcanti, 2018)
			CCA lateral-flow strip POC-RDT	(Peralta and Cavalcanti, 2018)
		Circulating anodic antigen (CAA)	ELISA	(Peralta and Cavalcanti, 2018)
			Up converted phosphor-lateral flow CAA assay	(Corstjens *et al*., 2014; Knopp *et al*., 2015; de Dood *et al*., 2018)
	*S. japonicum* (adult stage)	Circulating cathodic antigen (CCA)	CCA lateral-flow strip POC-RDT	(van Dam *et al*., 2015*a*,*b*)
		Circulating anodic antigen (CAA)	Up converted phosphor-lateral flow CAA assay	(van Dam *et al*., 2015*a*,*b*)
	*S. mekongi* (adult stage)	Circulating cathodic antigen (CCA)	CCA lateral-flow strip POC-RDT	(van Dam *et al*., 2015*a*,*b*; Vonghachack *et al*., 2017)
		Circulating anodic antigen (CAA)	Up converted phosphor-lateral flow CAA assay	(van Dam *et al*., 2015*a*,*b*; Vonghachack *et al*., 2017)
	*L. loa* (microfilariae larval stage)	LOAG_16297 protein	Nanobore reversed-phased liquid chromatography-tandem mass spectrometry (RPLC-MS/MS)	(Drame *et al*., 2016)
	*O. volvulus* (microfilariae larval stage)	Unspecified filarial larval antigen	POC-RDT dipstick assay	(Ayong *et al*., 2005)
Lymphatic system (adult stage/L3 larval stage) and/or circulatory system (microfilariae larval stage)	*W. bancrofti* (life- stage not specified)	Unspecified filarial antigen	Inhibition ELISA	(Chenthamarakshan *et al*., 1996)
			Sandwich ELISA	(Huijun *et al*., 1987)
			Double antibody sandwich ELISA	(Ramaprasad *et al*., 1988)
			Two-site immunoradiometric assay	(Henry *et al*., 1987)
			Counterimmuno-electrophoresis (CIEP)	(Weil *et al*., 1986)
		Urinary filarial antigen UFAC_2_	SDS-PAGE and immunoblotting	(Chenthamarakshan *et al*., 1993)
	*B. malayi* (life- stage not specified)	Unspecified filarial antigen	Sandwich ELISA	(Huijun *et al*., 1987)
Subcutaneous tissue	*O. volvulus* (adult stage)	Unspecified filarial antigen	Lateral-flow strip POC-RDT	(Ayong *et al*., 2005)
Central nervous system	*T. solium* (larval stage)	Cysticercal antigen	B158/B60 Urine Ag-ELISA	(Mwape *et al*., 2011)
		Secretory-excretory antigen	Monoclonal antibody Ag-ELISA	(Castillo *et al*., 2009)
		Unspecified *T. solium* antigen	Capture ELISA	(Paredes *et al*., 2016)
Liver	*O. viverrini* (adult stage)	Excretory-secretory antigen	Modified ELISA (Urine OV-ES) Assay	(Worasith *et al*., 2015)

The reliability of urine-antigen POC-RDTs when used in low-endemicity settings or when assessing individuals with low-intensity infections that may give unclear ‘trace’ results is, however, disputed (Coelho *et al*., 2016; Peralta and Cavalcanti, 2018). Recent meta-analyses suggest that, although more rapid and sensitive than stool-microscopy, under these circumstances' targeting the schistosome urine circulating cathodic antigen (CCA) by use of the CCA-POC-RDT is not sufficiently sensitive to reliably detect *S. mansoni* infection at the individual level (Danso-Appiah *et al*., 2016). It has been concluded that because of their low-cost, ease of use and patient-compliance, the CCA-POC-RDT may serve as a useful tool for disease-prevalence mapping and monitoring of control programmes relevant to *S. mansoni* in high- and medium-endemicity settings. In low-endemicity settings, however, when highly sensitive diagnostics capable of detecting low-intensity infections with a range of helminth species at the individual level are required, alternative and more sensitive assays are needed.

Revisions in assay protocols can improve the sensitivity and specificity of RDT's capable of detecting schistosome-urine-antigens beyond that of even lab-based ELISA assays, even in light-infections (Coelho *et al*., 2016; Kamel *et al*., 2019). Recently, the development of an up-converting phosphor lateral-flow (UCP-LF) assay targeting transrenal circulating anodic antigens (CAA) has shown that diagnosis of ultra-light schistosome infections through urine-CAA detection is possible (Corstjens *et al*., 2014; van Dam *et al*., 2015*a*,*b*). The genus-specific assay has shown extremely high sensitivity for the detection of *S. haemotobium, S. mansoni, S. japonicum* and *S. mekongi* urine-CAA, even in low-endemicity settings (Corstjens *et al*., 2014; van Dam *et al*., 2015*a*,*b*; Knopp *et al*., 2015; de Dood *et al*., 2018; Sousa *et al*., 2019). Further to its high-specificity, the UCP-LF CAA assay offers additional advantages over urine- and stool- microscopy in that the UPC-LF CAA is much higher-throughput and that only urine sampling is required to diagnose both urogenital and intestinal schistosomiasis (Knopp *et al*., 2015; Corstjens *et al*., 2014). Though treatment of both forms of schistosomiasis is identical, (40 mg praziquantel per kg body weight), if using this assay in areas co-endemic for both *S. haemotobium* and *S. mansoni*, additional steps would be required to diagnose species-specific infection, associated pathologies, cure rates and drug efficacies.

Although not yet fully suited for point-of-care use, the UCP-LF CAA assay requires only a reliable source of electricity, simple centrifugation facilities and pipetting capacities; offering a high-throughput and highly-sensitive means of diagnosing schistosomiasis through urine sampling whilst requiring lesser-equipped laboratory infrastructure than conventional immunodiagnostic assays (Knopp *et al*., 2015; Sousa *et al*., 2019). As the assay is also currently too expensive for commercial and routine use in schistosomiasis control programmes, efforts to develop a less expensive, rapid and simple-to-use CAA-POC-RDT that retains the UCP-LF CAA's high-sensitivity have begun (Knopp *et al*., 2015). Until then, it has been suggested that the existing assay could be used as a robust means of confirming or refuting indecisive test results at the individual level given by alternative, less sensitive but more field-appropriate, methods (de Dood *et al*., 2018).

Additional advancements in antigen-detecting immunodiagnostic POC-RDTs include the development of a lateral flow immunochromatographic test strip capable of detecting circulating *S. mansoni* antigen (CSA) within the urine using colloidal gold and mesoporous silica nanoparticles (Kamel *et al*., 2019). Though currently adapted only for diagnosis of infection with *S. mansoni*, these rapid and field-applicable test strips showed extremely high sensitivity when used to assess patients burdened with light infections and were even found to provide a more sensitive diagnosis than the conventional lab-based sandwich ELISA. Further assessment and validation of these RDT test strips, as well as adaptation for detection of other helminth-species urine-antigens, is encouraged.

It should be noted that the diagnostic potential of targeting any helminth-derived antigen through urine sampling will greatly depend on whether or not that antigen is expelled in the urine. Blood-circulating antigens with a high molecular mass may be too large to pass from the glomerular capillaries into the glomerular capsule and onto the bladder, and even of those that do, some will undoubtedly degrade into smaller products not recognised by monoclonal antibodies prior to diagnosis (Chanteau *et al*., 1994). Moreover, some helminth antigens may not be expelled in the urine because of that helminth species' bodily habitat. The adult form of *Ascaris lumbricoides* and various species of cestode, for example, reside within the gastrointestinal lumen and so do not directly interact with circulating blood (Lamberton and Jourdan, 2015).

Additionally, as with antibodies, any transrenal antigens targeted for diagnostic purposes will require assessment as to whether or not and to the degree with which they may cross-react with other antigens and/or other proteins expelled in the urine. Helminth-derived blood-circulating antigens from various genera of filarial nematode, for example, are known to cross-react in co-endemic areas; severely hampering the diagnostic efficacy of assays needed to identify infected individuals and provide safe treatment (Hertz *et al*., 2018). In addition, the *Schistosoma* CCA assay has been found to cross-react with antigens from other parasites, general inflammatory biomarkers and even metabolites expelled in the urine of pregnant women; also hampering diagnostic efficacy (van Dam *et al*., 1996; Utzinger *et al*., 2016).

## Detection of helminth-derived transrenal nucleic acid

Diagnosis *via* detection of helminth DNA expelled in the urine has many advantages beyond ease of sample procurement; it can be highly sensitive (trace levels of DNA can be detected), highly specific, parasite load can be quantified, assays can be high through-put and multiple species of parasitic helminth can be identified within one multiplex assay (Gordon *et al*., 2011; Phuphisut *et al*., 2014; Melchers *et al*., 2014). Possible further benefits include the early detection of anthelminthic drug resistance development, the ability to monitor helminth population genetic variation over time, relatively less arduous sample preparation when compared to blood, stool or tissue samples and the ability to detect pre-patent infections (Enk *et al*., 2010, 2012; Lamberton and Jourdan, 2015; Minetti *et al*., 2016).

Cell-Free DNA (cfDNA) has been defined as extracellular fragments of DNA found in bodily fluids or tissues, including the urine (Weerakoon *et al*., 2015, 2018; Weerakoon and McManus, 2016). It can be detected through use of nucleic acid amplification tests (NAATs), the more common of which include conventional polymerase chain reaction (PCR), nested PCR (nPCR) and quantitative or real-time PCR (qPCR/rtPCR) (Gordon *et al*., 2011; Verweij and Stensvold, 2014). Praised for their high sensitivity and specificity, NAATs are now becoming recognised as a more reliable means of helminth diagnosis than current gold standard and immunodiagnostic assays, particularly in low-intensity infections and even when targeting cfDNA expelled in the urine (Enk *et al*., 2012; Ibironke *et al*., 2012; Melchers *et al*., 2014; Lodh *et al*., 2016; Krolewiecki *et al*., 2018).

To date, using NAATs, numerous studies have evaluated the diagnostic efficacy of targeting transrenal cfDNA from helminths known to reside within a range of bodily habitats, all of which have reported higher sensitivity when compared to gold standard techniques (Table 4). Although the detectible presence of transrenal cfDNA has not yet been confirmed for all human-infecting parasitic helminths, as validated assays do currently exist for the detection of many helminth species' cfDNA in other bodily samples, adaptation of these to assess presence and diagnostic efficacy of helminth-derived cfDNA within the urine should be straightforward (Gordon *et al*., 2011; Minetti *et al*., 2016; Weerakoon and McManus, 2016). Of particular interest would be to determine the presence of transrenal *Onchocerca volvulus and Loa loa* cfDNA, given their subcutaneous and deep tissue habitats and understandable patient aversions to invasive skin-snip biopsies currently used to confirm onchocerciasis infection (Knopp *et al*., 2012). Of additional interest would be to determine the presence of transrenal cfDNA from *Trichuris trichiura* and hookworm parasites as, despite sharing their gastrointestinal tract habitat with *Ascaris lumbricoides* and cestodes, adult forms do interact with circulating blood (Jourdan *et al*., 2018). As with *A. lumbricoides* and cestode urine-antigen detection, detection of transrenal cfDNA from these helminths may be unlikely. Cell-free DNA from *Strongyloides stercoralis*, another gut-dwelling helminth, has been successfully detected in urine, though it is speculated this is due to tissue dissemination during larval-form autoinfection (Lodh *et al*., 2016).

**Table 4.** Helminth cfDNA detected within the urine and nucleic acid amplification test (NAAT) used.

Bodily habitat	Species	Antigen detected	Assay used	References
Circulatory System	*S. haematobium*	121 bp repeat fragment *Dra*1	PCR	(Shiff *et al*., 2011; Ibironke *et al*., 2012; Lodh *et al*., 2014)
			RPA	(Rostron *et al*., 2019)
		rDNA internal transcribed spacer (ITS) region	PCR	(Sandoval *et al*., 2006)
		77 bp fragment of internal transcribed spacer-2 (ITS2) sub-unit	PCR	(Aryeetey *et al*., 2013)
		Fragments of small subunit SSU rRNA	LAMP	(Bayoumi *et al*., 2016)
		Oligonucleotides: F3; B3; FIP; BIP (Hamburger *et al*., 2013)	LAMP	(Lodh *et al*., 2017)
		77 bp fragment of internal transcribed spacer-2 (ITS2) sub-unit	qPCR/rtPCR	(Melchers *et al*., 2014)
		Variable 267 bp region within cytochrome c oxidase subunit 1 (*cox1*) mitochondrial DNA	qPCR/rtPCR	(Sady *et al*., 2015)
	*S. mansoni*	rDNA internal transcribed spacer (ITS) region	PCR	(Sandoval *et al*., 2006)
		28S rDNA region	PCR	(Sandoval *et al*., 2006)
		110 bp region from highly repeated 121 bp fragment (Hamburger *et al*., 1991)	PCR	(Enk *et al*., 2012; Lodh *et al*., 2013, 2014)
		Oligonucleotides: F3; B3; FIP; BIP (Hamburger *et al*., 2013)	LAMP	(Lodh *et al*., 2017)
		Unnamed 650 bp mitochondrial *S. mansoni* minisatellite region	LAMP	(Fernández-Soto *et al*., 2019)
		Fragment of mitochondrial cytochrome c oxidase subunit 1 (CO1) gene	qPCR/rtPCR	(Sady *et al*., 2015)
	*S. japonicum*	Fragment of mitochondrial cytochrome c oxidase subunit 1 (CO1) gene	PCR	(Kato-Hayashi *et al*., 2015)
Lymphatic system (adult stage/L3 larval stage) and/or circulatory system (microfilariae larval stage)	*W. bancrofti*	Unnamed tandem-specific region (Kanjanavas *et al*., 2005)	PCR	(Ximenes *et al*., 2014)
		188 bp fragment from SspI repeat sequence	PCR	(Lucena *et al*., 1998)
Gastrointestinal tract (adult stage) and/or circulatory system (larval stage)	*S. stercoralis*	124 bp fragment from dispersed repeat sequence AY028262	PCR	(Lodh *et al*., 2016; Krolewiecki *et al*., 2018)
Central nervous system	*T. solium* (larval stage)	116 bp fragment from pTsol-9 gene	PCR	(Toribio *et al*., 2019)

Although clearly a highly sensitive method of confirming or refuting infection, many financial, logistical and methodological challenges must be overcome if NAATs are to replace current diagnostic standards, regardless of the bodily sample taken. Perhaps of primary concern are the high costs associated with NAATs, such as PCR and qPCR, when compared to current, less costly, gold standard assays. Expensive reagents, sophisticated equipment and remuneration of specialist technical staff all contribute to overall expenditure, again resulting in a diagnostic assay likely unaffordable to most health workers in resource-poor settings (Minetti *et al*., 2016). Another major challenge is the upscale and field-applicability of assays targeting cfDNA. In programmatic elimination settings where rapid and reliable detection of few infected individuals is crucial for disease elimination, diagnostic assays must be deployable at the point-of-care. Not only are conventional NAATs themselves currently unsuited for point-of-care use, but essential sample preparation steps, such as DNA extraction, that also require specific laboratory equipment and reagents, prevent the use of conventional NAATs anywhere lacking sophisticated laboratory infrastructure. In addition, to what extent helminth-derived cfDNA continues to be expelled in the urine after infection clearance is largely unknown and likely varies between parasite species' bodily habitat and degree of the previous infection.

The recently developed loop-mediated isothermal amplification (LAMP) assay shows promise for future point-of-care use; DNA fragments are amplified under isothermal conditions, negating the need for thermocycling equipment essential for PCR-based assays; the assay is rapid; results can be seen with the naked-eye; multiple pathogens can be targeted and detected using one assay run; assays can be carried out by non-specialist staff; reagents can be lyophilised and initial DNA extraction steps may be less laborious (Gordon *et al*., 2011; Zhang *et al*., 2014; Weerakoon and McManus, 2016; Bayoumi *et al*., 2016; Deng *et al*., 2019). To date, LAMP has been used to successfully detect and amplify helminth DNA from other bodily samples; *S. mansoni*-derived cfDNA in urine samples taken from experimentally infected mouse models; cfDNA of *Strongyloides venezuelensis* (a rodent-infecting species) in urine samples taken from experimentally infected rat models and *S. haematobium* DNA in human urine samples (Takagi *et al*., 2011; Fernández-Soto *et al*., 2014, 2016, 2019; Shiraho *et al*., 2016; Bayoumi *et al*., 2016; Lagatie *et al*., 2016). Despite these advantages, however, the LAMP assay does still require heat-blocks or waterbaths to heat reactions for long periods; often up to 2 hours. A reliable source of electricity is therefore still essential. Furthermore, unlike qPCR/rtPCR, LAMP assays are only semi-quantitative, meaning estimations of infection intensity are subjective and may vary between personnel; individual assays are low-throughput; amplified fragments cannot be sequenced, preventing the monitoring of genetic variation in populations over time and ambiguity exists around LAMP sensitivity when compared to alternative PCR-based approaches (Verweij and Stensvold, 2014; Zhang *et al*., 2014; Minetti *et al*., 2016).

To overcome many of the logistical and methodological challenges presented by PCR-based diagnostics, the point-of-care recombinase DNA-polymerase amplification (RPA) assay has also recently been developed and has been used to successfully detect and amplify *S. japonicum* and *F. hepatica* cfDNA in human stool samples, as well as *S. haematobium* DNA within human urine samples (Piepenburg *et al*., 2006; Sun *et al*., 2016; Xing *et al*., 2017; Cabada *et al*., 2017; Li *et al*., 2019; Rostron *et al*., 2019). Assays to detect *S. mansoni* DNA by the use of the RPA have also recently been developed, though these have not yet been tested on clinical samples (Poulton and Webster, 2018).

Capable of detecting even trace levels of DNA, the RPA provides a promising means of reliably detecting ultra-light levels of infection in low-endemic areas (Rosser *et al*., 2015; Lai *et al*., 2017; Poulton and Webster, 2018; Rostron *et al*., 2019). In addition, the assay itself offers many advantages over PCR and qPCR in terms of its methodology and potential use at the point-of-care (Aryeetey *et al*., 2013; Lodh *et al*., 2014; Sady *et al*., 2015; Minetti *et al*., 2016).

Firstly, assay reactions take place within a robust, hand-held, easily programmed, portable and battery-powered device, omitting the need for specialist technical personnel and sophisticated laboratory infrastructure. Moreover, assay results can be easily interpreted using the same device or, alternatively, *via* simple-to-use and low-cost lateral-flow immunoassay strips; both omitting the need for sophisticated and delicate readout equipment (Rosser *et al*., 2015; Xing *et al*., 2017; Poulton and Webster, 2018; Rostron *et al*., 2019).

Assay reactions are also isothermal; optimal amplification occurs within 25°C – 42°C with use of the device's battery-powered heater, though testing can take place at ambient temperature in some endemic areas, or even at body temperature (Crannell *et al*., 2014; Kersting *et al*., 2014; Minetti *et al*., 2016). Reaction time at reduced temperatures is, however, prolonged. Isothermal reactions not only negate the need for thermocycling equipment that may only amplify specific DNA strands based on cycle conditions within one cycle run but also allow for the detection and amplification of DNA from multiple helminth species or even other pathogens, e.g. malaria or intestinal protozoa, using only different primer combinations, within the same assay run (Crannell *et al*., 2016). Additionally, and unlike LAMP amplicons, RPA amplicons can be sequenced, allowing the monitoring of genetic variation in populations over time (Oyola *et al*., 2012).

Another advantage of RPA over PCR-based techniques is assay runtime; results can often be seen within 30 min of urine sample procurement as purification of total DNA from urine is not required (Kersting *et al*., 2014; Krõlov *et al*., 2014; Rosser *et al*., 2015; Rostron *et al*., 2019). Therefore, sample preparation is also less laborious and more field-applicable than these alternative DNA amplification methods as only crude preparations are needed. In addition, assay reagents can be lyophilised for easy transportation and can be stored without refrigeration even in tropical ambient temperatures for several weeks (Crannell *et al*., 2014; Oriero *et al*., 2015).

The RPA assay is, however, much higher in cost when compared to alternative DNA amplification approaches. Estimated to cost between 4 USD$ and 5 USD$ per sample, the assay is currently too expensive for routine use in population-level control programmes within endemic areas (Minetti *et al*., 2016). With further development, adaptation and uptake of the assay, however, the cost per assay sample is very likely to decrease in the near future (Rosser *et al*., 2015).

Further drawbacks to RPA include, like LAMP, the assay's low throughput when compared to alternative PCR-based methods, though this can be resolved through manufacture of larger-capacity devices. In addition, again like LAMP results, RPA results are only semi-quantitative, making estimations of infection intensity less reliable. As such, the assay in its current form may be best suited for small sample sizes and individual test-and-treat scenarios in low-endemicity settings.

Despite these disadvantages, the RPA is an extremely promising means of rapid, straightforward and sensitive diagnosis at the point-of-care in low-endemicity settings. Further development and validation of the RPA assay for use in diagnosing helminthiases using non-invasive urine sampling, is therefore recommended.

## Novel biomarkers

Proteomic and metabolomic technologies can be used to screen bodily samples, including urine, to identify novel biomarkers that may potentially be used for diagnostic purposes. Using liquid chromatography and mass spectrometry, for example, it was recently reported that as many as 31 *Schistosoma*-derived proteins were differently abundant within the urine of patients infected with *S. haematobium* when compared to an uninfected control group and so may be used to detect active infection (Onile *et al*., 2017). Here, it was also suggested that the presence and abundance of some transrenal host-derived proteins such as human growth/differentiation factor 15 (GDF15), upregulated in response to organ damage, may even provide a reliable means of determining disease severity and infection intensity, and so should be further evaluated.

Eosinophil cationic protein (ECP), involved in the body's immune response to foreign pathogens, has also been found to be significantly elevated in the urine of individuals infected with a variety of helminth species including *S. haematobium, S. mansoni, O. volvulus*, *W. bancrofti*, and hookworm (Tischendorf *et al*., 1999; Tischendorf *et al*., 2000; Klion and Nutman, 2004; Fayez *et al*., 2010; Asuming-Brempong *et al*., 2015). In addition to GDF15, when assessing the use of transrenal ECP as a biomarker for infection with *S. haematobium*, a positive correlation between expelled ECP and urine egg count was found, suggesting urinal ECP may too increase with infection intensity and may therefore potentially be used to assess disease severity and worm burden (Leutscher *et al*., 2008; Leutscher *et al*., 2000). These findings have since been replicated not only in *S. haematobium*, but also in *S. mansoni* infections (Asuming-Brempong *et al*., 2015). More recently, ECP levels in serum samples taken from individuals infected with hookworm have also been shown to positively correlate with infection intensity (Amoani *et al*., 2019). Repeated assessment using lesser-invasive urine samples was recommended.

Liquid chromatography has also been used in conjunction with infrared spectrophotometry to screen urine for expelled metabolites associated with helminth infections. Using this approach, it was reported that two metabolites, 2-methyl-butyramide and 2-methyl-valeramide, can be detected within the urine of individuals infected with *Ascaris* (Hall and Romanova., 1990). These findings have, however, recently been contested after neither of metabolite was detected in Indonesian individuals harbouring active *Ascaris* infections (Lagatie *et al*., 2017). In addition to liquid chromatography, nuclear magnetic resonance (NMR) spectroscopy has been used to screen urine samples taken from mice experimentally infected with *S. mansoni* for expelled metabolites (Wang *et al*., 2004). A range of transrenal metabolites was associated with active infection and so warrant further exploration in human urine samples and in other helminth species infections.

Newly discovered transrenal biomarkers with the potential to indicate active infection, parasite burden and morbidity status may help to inform and shape future point-of-care diagnostic tools. The continued use of proteomic and metabolomic technologies for biomarker discovery is therefore strongly encouraged.

## Discussion

Rapid, simple-to-use and low-cost diagnostic tools, deployable at the point-of-care and reliable in low-endemicity near-elimination settings, are urgently needed to help facilitate the elimination of debilitating parasitic helminth diseases. Current WHO-recommended gold standard assays do not meet these criteria and typically require invasive and potentially hazardous bodily samples. Many of these criteria are, however, met by non-invasive urine-based diagnostic assays capable of detecting a range of parasitic helminth species.

Macroscopic and microscopic changes to urine are not adequately sensitive to detect urogenital schistosomiasis in light-infections, preventing their use in near-elimination settings. In addition, although anti-helminth antibodies from a range of helminth species can be detected within the urine with high sensitivity, technical, financial and logistical challenges impede the reliability and routine use of urine-antibody diagnostic assays in helminth control programmes.

POC-RDT devices capable of detecting transrenal helminth-derived antigens may offer a simple, rapid, sensitive and low-cost diagnostic format at the point-of-care in high- and medium-prevalence settings. Although not currently adequately sensitive in low-endemicity settings or at the individual-level in patients burdened with light-infections, technological advancements and protocol revisions show promise for future improvements in POC-RDT diagnostic sensitivity that may facilitate their use in low-endemicity near-elimination settings.

Targeting transrenal helminth cfDNA is extremely sensitive and specific even in low-endemicity settings. The majority of assays capable of detecting urine-cfDNA are, however, both unsuited for point-of-care use and unaffordable to most control-programmes in disease-endemic areas. The recently developed LAMP and RPA assays may offer a promising, reliable and field-deployable means of detecting helminth-derived urine-cfDNA and, through further research, development and validation is needed before their routine application in disease-endemic areas, these assays have the potential for reliable test-and-treat use in low-endemicity near-elimination settings to rapidly identify lightly infected individuals capable of maintaining disease transmission.

The majority of current literature concerned with diagnosing helminthiases through urine sampling focuses primarily on the diagnosis of urogenital and intestinal schistosomiasis. As outlined here, many other helminth species, from a range of boldily habitats, can be detected through non-invasive urine sampling, particularly *via* targeting transrenal helminth-derived antigens and cell-free DNA. As such, the following research priorities are proposed:

- To ascertain the detectable presence of transrenal antigens and cfDNA from all of the major human-infecting parasitic helminth species.
- To determine any potential cross-reactivity of transrenal helminth-derived antigens with other antigens and/or proteins expelled in the urine and decipher how long helminth-derived cfDNA continues to be expelled within the urine after infection clearance.
- Further development and validation of rapid diagnostic tests and field-deployable assays suitable for point-of-care use and able to reliably detect trace levels of helminth-derived urine-antigens and cfDNA known to be expelled in the urine. Assay assessment should not use traditional and unreliable gold standard techniques as reference, but rather more sensitive and specific assays, such as qPCR, as reference.

Despite the high financial costs associated with developing, validating and implementing novel diagnostic tools, the programmatic and economic benefits, as well as the health benefits to those in disease-endemic areas, gained from improved diagnostics capable of detecting even trace infections at the point-of-care will very likely outweigh any initial expenditure (Turner *et al*., 2017). The continued investment in and development of reliable, low-cost and non-invasive urine-based diagnostic assays deployable at the point-of-care is therefore highly encouraged.

## Concluding remarks

Sensitive and specific diagnosis of many major parasitic helminthiases at the point-of-care is likely possible through non-invasive urine sampling. Though further development, assessment and validation are needed before their routine use in control programmes, low-cost and rapid assays capable of detecting transrenal helminth-derived antigens and cell-free DNA show promise for future use at the point-of-care in high-, medium- and even low-endemicity elimination settings. Ultimately, however, until these techniques are more affordable and easily implemented, less-reliable assays that require more invasive bodily samples will remain the diagnostic standard.
